# Antioxidant Properties Mediate Nephroprotective and Hepatoprotective Activity of Essential Oil and Hydro-Alcoholic Extract of the High-Altitude Plant *Skimmia anquetilia*

**DOI:** 10.3390/antiox12061167

**Published:** 2023-05-28

**Authors:** Neha Kukreti, Havagiray R. Chitme, Vinay K. Varshney, Basel A. Abdel-Wahab, Masood Medleri Khateeb, Mohammed Shafiuddin Habeeb

**Affiliations:** 1 Faculty of Pharmacy, DIT University, Dehradun 248009, India; 1000013858@dit.edu.in; 2 Chemistry & Bioprospecting Division, Forest Research Institute, Dehradun 248006, India; varshneyvk@icfre.org; 3 Department of Pharmacology, College of Pharmacy, Najran University, Najran P.O. Box 1988, Saudi Arabia; babdelnaem@nu.edu.sa (B.A.A.-W.); mmkhateeb@nu.edu.sa (M.M.K.); mshabeeb@nu.edu.sa (M.S.H.)

**Keywords:** hepatoprotective, antioxidant, anti-haemolytic, phenolic, flavonoid, DPPH

## Abstract

There are many high-altitude plants such as *Skimmia anquetilia* that are unexplored for their possible medicinal values. The present study was conducted to examine the antioxidant activities of *Skimmia anquetilia* (SA) using in vitro and in vivo models. The SA hydro-alcoholic extracts were investigated using LC-MS for their chemical constituents. The essential oil and hydro-alcoholic extracts of SA were evaluated for pharmacological properties. The antioxidant properties were evaluated using in vitro DPPH, reducing power, cupric reducing antioxidant power, and metal chelating assays. The anti-hemolytic activity was carried out using a human blood sample. The in vivo antioxidant activities were evaluated using CCL_4_-induced hepatotoxicity and nephrotoxicity assay. The in vivo evaluation included histopathological examination, tissue biochemical evaluation such as the kidney function test, catalase activity, reduced glutathione activity, and lipid peroxidation estimation. The phytochemical investigation showed that the hydro-alcoholic extract contains multiple important active constituents such as L-carnosine, acacetin, linoleic acid, leucylleucyl tyrosine, esculin sesquihydrate, etc., similar to the components of SA essential oil reported in a previous study. The high amount of total phenolic content (TPC) and total flavonoid content (TFC) reflect (*p* < 0.001) a high level of reducing power, cupric reducing, and metal chelating properties. This significantly (*p* < 0.001) inhibited enlargement of the liver, with a significant reduction in ALT (*p* < 0.01) and AST (*p* < 0.001). Highly significant improvement in the functioning of the kidney was noted using the blood urea and creatinine (*p* < 0.001) levels. Tissue-based activities showed a major rise in catalase, reduced glutathione, and reduced lipid peroxidation activities. We conclude from this study that the occurrence of a high quantity of flavonoid and phenolic contents had strong antioxidant properties, leading to hepatoprotective and nephroprotective activity. Further active constituent-specific activities should be evaluated.

## 1. Introduction

ROS (reactive oxygen species) are formed as secondary products during aerobic metabolism in living organisms such as superoxide (O_2_), H_2_O_2_ (hydrogen peroxide), singlet oxygen (O), hypochlorous acid (HOCl), hydroperoxyl (HOO•), peroxyl (ROO•), and hydroxyl (HO•) [1,2]. These radicals are typically regarded as an integral component of aerobic metabolism, and the rates at which they are generated and removed are almost equal in normal conditions [3,4]. Any imbalance in the production and removal of free radicals would cause oxidative stress, which leads to irreversible adverse alterations that result in cell death (apoptosis), ageing, and oxidation occurring in cell components [2,5]. Majorly, the liver plays an important multiple function to balance actions in the body including the disposition and detoxification of endogenous and foreign compounds. These free radicals are also known to damage cells by peroxiding unsaturated fatty acids, denaturing proteins, and reacting with nucleic acids and carbohydrates [6]. It is also known that the respiratory oxidative phosphorylation cycle can damage proteins, lipids, DNA, RNA, and carbohydrates leading to the emergence of these harmful agents. The oxidative stress cycle is well known for the pathogenesis of several diseases that include carcinogenesis, ulcerative colitis, cardiovascular diseases, and autoimmune and neurological degenerative diseases [1,4,5,7]. 

In general, the industrial solvent carbon tetrachloride (CCl_4_) can cause hepatocyte damage in animal models by enhancing the endoplasmic reticulum’s ability to break down into highly reactive trichloromethyl (CCl_3_) and trichloro-methylperoxy (OOCCl_3_) radicals. The liver cells’ unsaturated fatty acids react with free radicals in the presence of phospholipids, resulting in damage to hepatocytes [8]. The CCl_4_-induced hepatotoxicity is the commonly preferred method for in vivo antioxidant studies due to its reproducibility and acceptability. Butylated-hydroxytoluene (BHT), propyl gallate, butylated-hydroxyanisole (BHA), and tert-butylhydroquinone are frequently utilized as synthetic antioxidants [4]. There are endless sources of physiologically active chemicals and medicinal metabolites found in plants that are used in conventional medicines and proven to be safe and effective [9]. They have been used for many centuries throughout the world to cure/manage a number of ailments in both humans and animals because of the presence of active chemical components that induce therapeutic physiological effects. Indeed, they are the major source and inspiration for new drug discovery and development, especially for complex conditions [10,11].

Plants include large amounts of phenolic, carotenoid, flavonoid, anthocyanin derivatives, vitamins, unsaturated fatty acids, enzymes, and cofactors, all of which have the ability to stabilize free radicals [3,12]. Polyphenols and terpenes, which are primary aromatic ingredients, make up the biggest category of bioactive substances in plants and are crucial in medicine [9,12]. Polyphenols (flavonoids and phenolic acids) have an antioxidant and anti-inflammatory potential and are frequently used in the human diet to interfere with signalling pathways and gene expression and also activate enzymes that cause the elimination of an oxidant species; they are also used for various defence pathways against naturally occurring skin damage and prevent skin cancer [11,13,14]. These antioxidants are proven to stabilize and scavenge free radicals, preventing oxidative damage to an organism [15]. These antioxidants, also known as nutraceuticals, prevent the production of metal radicals, inhibit lipid peroxidation, promote gene expression, and repair damaged molecules [16]. 

The GCMS study of essential oils from SA reported that they are enriched with total phenolic compounds [17,18]. Results in Table 1 are based on the LC/MS study of SA hydro-alcoholic extract. γ-terpinene and α-selinene are the important antioxidant constituents of *Skimmia anquetilia* (*SA*) essential oil, as shown in Table 2. *SA* is commonly known as Kedarpatti and belongs to the Rutaceae family. It is an evergreen shrub and has aromatic essential oils in the height of the Himalayan range in Nepal, Afghanistan, India, and Pakistan. The SA plant has bright red and ovoid shape berries. According to a literature survey, SA has been used for various pharmacological activities such as anti-arthritic, antibacterial, anti-inflammatory, antifeedant, and antioxidant [17]. Therefore, the objective of the present study was to evaluate the antioxidant-mediated hepatoprotective and nephroprotective effects of *Skimmia anquetilia* (*SA*) using in vitro and in vivo experimental methods.

## 2. Materials and Methods

### 2.1. Chemicals Required

Sodium carbonate, quercetin, aluminium chloride (AlCl_3_), Folin–Ciocalteu reagent, methanol, DPPH (2, 2-diphenyl-1-picrylhydrazyl), thiobarbituric acid, gallic acid, sodium–potassium tartrate, and Ellman’s reagent were procured from Hi-media. Iron chloride hexahydrate, sodium acetate trihydrate 99% (Darmstadt, Germany), and double distilled water were used in this study.

### 2.2. Sample Collection

*SA* leaves were collected from the Puroula district (Uttarakhand, India) and deposited at the Botanical Survey of India (BSI) with a voucher specimen Tech./Herb (Ident.)/2022-23/824 (Acc. No. 1259). The specimens were authenticated by Dr. S.K. Singh, Scientist-E/HOO from the Botanical Survey of India (BSI), Dehradun, India, as *Skimmia anquetilia N.P.Taylor and Airy Shaw*, Family: Rutaceae and Order: Sapindales.

### 2.3. Hydro-Alcoholic Extraction

In total, 50 g of powdered *SA leaves* in a ratio 1:10 of 25% hydro-alcoholic (MeOH) solvent was kept in an automated ultrasonic bath at 45 ± 1 °C. The beaker was closed with aluminium foil to avoid and minimize methanol evaporation. The solution was filtered, the residue was blended in a given quantity of the 25% hydro-alcoholic solvent, and then the process was repeated until the hydro-alcoholic extracts became clear. The filtered solution was then evaporated using a rotary evaporator at 45 °C until we obtained a semisolid mass. The semisolid mass was dried in a freeze dryer at −20 °C. The freeze-dried preparation was kept in an airtight container at 4 °C until needed for analysis and study. The percentage yield of the hydro-alcoholic extract was calculated to be 20.58% [19].

### 2.4. Extraction of Oil

Essential oil was obtained using the hydro-distillation method. In total, 50 g of fresh leaves from *SA* were added to a round bottom flask and placed onto the distillation unit. Steam was evaporated in an upward direction into the biomass flask, condensed in a chiller, and collected in receiving flask. According to density, the separation of water–oil occurred in the receiving flask for collection. The oil sample was passed in anhydrous sodium sulphate (Na_2_SO_4_) to remove the water molecules and then was stored in sealed vials at 4 °C [20]. The percentage yield of the oil was calculated to be 1.9%.

### 2.5. LC/MS Technique

LC/MS analysis of the hydro-alcoholic extract was performed using XEVO-TQD#QCA1232 coupled with a Waters Alliance e2695/HPLC-TQD Mass spectrometer and a column SUNFIRE C18 (250 × 2.1, 2.6 µm). A liquid sample was used in this analysis. The hydro-alcoholic extract was dissolved in methanol (2 mg/mL). The eluents used for LC/MS were acetonitrile (5%) and ammonium formate (95%). The mass spectra of compounds present in the hydro-alcoholic extract were identified with http://spectra.psc.riken.jp/menta.cgi/respect/index (accessed on 23 November 2022) and the published literature.

### 2.6. Total Phenolic Content (TPC)

TPCs were estimated using a modified method to analyse the Folin–Ciocalteu level. We added 1000 µL of hydro-alcoholic extract (1000 μg/mL) in 5000 µL of Folin–Ciocalteu reagent (10%), vortexed the sample, and then added 4 mL of Na_2_CO_3_ (2%). Methanol was used as a control, and the samples were incubated for 1.5 h at room temperature. The absorbance was measured using a UV-Vis spectrophotometer (Biogen) at 765 nm. The phenolic content was determined using a plot showing the calibration curve of gallic acid (200–400 μg/mL). TPC was calculated as the mg of gallic acid equivalent (GAE) per gram of dry weight of extract [18]. The equation for the calibration curve was:

Y = 0.0056x + 0.0766

R^2^ = 0.9981

### 2.7. Total Flavonoid Content (TFC) 

First, a calibration curve for quercetin was prepared in methanol (50–400 μg/mL). Then, Na-K tartrate (100 μL), AlCl_3_ (100 μL of 10% *w*/*v*), and distilled water (2.8 mL) were blended to a 250 µL aliquot of each sample (hydro-alcoholic extract) at 500 μg/mL and 1000 μg/mL. Thereafter, we followed the steps mentioned in previously published studies [21,22].

### 2.8. Antioxidant Activity

#### 2.8.1. In Vitro Activity

DPPH Radical Scavenging Assay: First, 1.0 mL of different concentrations of SAE and ascorbic acid (10–100 mg/mL) and SAEO (100, 500, 1000, and 2000 µg/mL) were added into a test tube. Then, DPPH in methanol (2.0 mL, 0.1 mM) was mixed in the hydro-alcoholic extract, oil, and standard sample and incubated at 37 °C for 30 min. The absorbance was recorded at 517 nm. All results were calculated using the following formula:

$$\mathrm{DPPH}\;\mathrm{radical}\;\mathrm{scavenging}\;\left(\%\right)=\left[\frac{1-A_{s}}{A_{0}}\right]\times 100$$

where A_s_ = the absorbance of the sample and A_0_ = the absorbance of the control [23]. 

Fe^3+^ Reducing power Assay: In this assay, the reduction from Fe^3+^ to Fe^2+^ is recorded using the absorbance of a blue complex [24]. First, 1 mL of hydro-alcoholic extract or oil (20–100 µg/mL) was added to 2.5 mL of 0.2 M sodium phosphate buffer at pH 6.6, 2.5 mL of 1% *w*/*v* potassium ferricyanide [K_3_Fe(CN)_6_], and 1 mL of distilled water. The mixture was heated at 50 °C in a water bath and incubated for 20 min. Afterwards, TCA (2.5 mL at 10%) was added to the reaction mixture, and then the mixture was centrifuged at 1000× *g* for 10 min. A 2.5 mL sample of fluid was removed from the supernatant and then mixed with 0.5 mL of 0.1% *w*/*v* FeCl_3_ and distilled water (2.5 mL). The tests were carried out in triplicate, and the absorbance of the reaction mixtures was measured at 700 nm using a UV-Vis spectrophotometer (Biogen, Cambridge, MA, USA). Ascorbic acid was used as the standard. A greater reductive potential was indicated by the higher absorbance of the reaction mixture [14].

Cupric Reducing Antioxidant Power (CUPRAC) Assay: First, 100 µL of SAE and SAEO (20–100 µg/mL) was added with 1 mL of copper chloride solution (10 mMolar)–neocuproine (7.5 mM) alcoholic solution in 99.9% methanol and 1 M of ammonium acetate buffer (pH 7.0) solution as well as 1 mL of distilled water to make final volume 4.1 mL. Thereafter, the samples were incubated for 30 min at 37 °C, and the absorbance was observed against the reagent blank at 450 nm. A standard curve was prepared using ascorbic acid (20–100 µg/mL). The results were expressed as 𝜇mol ascorbic acid /g [25]. 

Metal chelating Assay: To prepare 2 ml, we first added 1 ml of various concentrations of hydro-alcoholic extract and oil (20–100 µg/mL) to 0.5 mL of 2.5 mM FeCl_2_ and de-ionized water, left the mixture to stand for 5 min, and then added 0.5 mL ferrozine (5 mM). Ferrozine forms a stable magenta-coloured complex species when reacted with a divalent iron. At the same concentration, ascorbic acid was treated as a standard control. Afterwards, the reaction mixture was centrifuged and incubated for 10 min at 37 °C. The Fe^2+^–Ferrozine complex absorbance was observed at 562 nm using a UV-Vis spectrophotometer. The mixtures were observed in triplicate. The chelation activity of the hydro-alcoholic extract and oil was evaluated as follows:

$$\mathrm{Chelating}\;\mathrm{rate}\;\left(\%\right)\;\mathrm{of}\;a\;\mathrm{sample}=\left[\left(A_{0}-A_{1}\right)÷A_{0}\right]\times 100\;$$

where A_1_ and A_0_ were an absorbance of the blank and the sample [26,27].

#### 2.8.2. In Vivo Antioxidant Study

Experimental Animals: Male Swiss albino mice (35–50 g) were purchased from NIB Ghaziabad, India. Animals were housed in diurnal lighting conditions (12 h/12 h) and provided standard polypropylene animal cages at 22 ± 2 °C. This study was conducted after obtaining approval from the Institutional Animal Ethics Committee (approval no. DITU/IAEC/21-22/07-07).

Acute Oral toxicity Studies: A female mice model was used to calculate the LD_50_ values according to the OECD-423 guideline for acute oral toxicity studies. *SA* hydro-alcoholic extract and oil were administered orally (500, 1000, and 2000 mg/kg) and individually using an oral feeding needle [28]. Each dose selected was administered to a group of three animals.

Experiment of Carbon tetrachloride-induced hepatotoxicity: A total of 45 mice were allocated into 9 groups with each group having 5 mice. A dose of the treated sample and the standard drug was given in oral single dose for 21 days [29,30,31]. Group I Control (vehicle): Received 0.9% saline up to 25 mL/kg of body wt. Group II (CCl_4_ induced): Received CCl_4_ + olive oil in a 1:1 *v/v* up to 1.5 mL/kg of body wt. ip. Group III (standard group): Received CCl_4_ + vitamin E capsules up to 40 mg/kg. Group IV (low dose of hydro-alcoholic extract): Received CCl_4_ + 25% hydro-alcoholic SA extract up to 100 mg/kg. Group V (moderate dose of hydro-alcoholic extract): Received CCl_4_ + 25% hydro-alcoholic SA extract up to 200 mg/kg. Group VI (high dose of hydro-alcoholic extract): Received CCl_4_ + 25% hydro-alcoholic SA extract up to 400 mg/kg. Group VII (low dose of oil): Received CCl_4_ + SA essential oil (daily single dose up to 100 mg/kg, p.o.) for 21st days. Group VIII (moderate dose of oil): Received CCl_4_ + SA essential oil up to 200 mg/kg. Group IX (high dose of oil): Received CCl_4_ + SA essential oil up to 400 mg/kg.

On the 20th day, 1.5 mL/kg dose of CCl_4_ in olive oil (1:1 *v*/*v*) was administered intraperitoneally (i.p.) to groups II to IX after 1 h of dosing with the standard drug, hydro-alcoholic extracts, and oils, whereas group I received 10 mL/kg of olive oil (i.p.) only. After 24 h, blood samples were collected under mild anaesthesia and then all animals were euthanized using cervical dislocation and the liver was excised for biochemical analysis. The body weight of mice in all groups was recorded on the 1st and 22nd day, which was used to calculate the change in body weight that occurred due to the treatment. Liver weight was also calculated to determine the drug’s effect on mouse morphology and physiology.

Organ index: The organ index was measured using the below formula [29,30,31]:

Organ index = (liver weight/body weight) × 100%.

Evaluation of Hepatoprotective and nephroprotective Activity: Biochemical indicators for hepatic serum glutamic–oxaloacetic transaminase and serum glutamic pyruvic transaminase (SGOT and SGPT) and kidney (urea and creatinine) were used to measure acute liver and kidney injury. Serum SGOT and SGPT (ALT and AST) and urea and creatinine levels were measured using an Erba diagnostic kit. A sample of blood was collected from the retro-orbital route and mixed in anticoagulant (EDTA) tubes. The collected blood was centrifuged (3000 rpm, 15 min), and the serum samples were stored in a deep freezer at −80 °C until the determination of biochemical and immunological parameters [32,33].

Histopathological Analysis: Liver tissues were excised, cleaned with PBS at pH 7.4, and cut into two pieces. One section was used for histopathological analysis (10% formalin), and another 1 g section was homogenized with 9 mL of PBS at pH 7.4 for in vivo analysis [34].

Liver tissue homogenization: Tissue homogenate fluid was cold centrifuged at 4 °C (10,000× *g*, 15 min). The supernatant fluid was collected in centrifuge tubes and stored in a deep freezer (−80 °C) until further analysis [35,36].

Tissue biochemical parameters: Catalase Activity (CAT): In brief, 50 µL of the tissue homogenate was added in PBS (2 mL, pH 7.0) and H_2_O_2_ (1 mL of 30 mM). The samples were incubated for 1 min, and CAT activity was recorded using a spectrophotometer at 240 nm. CAT was calculated as units per milligram of protein [37,38]. 

Reduced glutathione activity (GR): Reduced glutathione (GR) was estimated by mixing excised liver homogenate (1 mL) in an equal volume of TCA (10%). The mixture was incubated (5 min) and then cold centrifuged (2000 rpm for 10 min). Thereafter, this study was conducted as mentioned in previous studies. The absorbance of the mixture was measured at 412 nm, and the amount of reduced glutathione was calculated as µg/mg of protein [38,39].

Estimation of Lipid Peroxidation (LPO): LPO was estimated by adding 100 µL of tissue homogenate in 2 mL of 1:1:1 ratio of a reagent, which involved TBA (0.37%), HCl (0.25 N), and Trichloro acetic acid (15%), and then keeping the mixture for 15 min in a water bath. Cool, centrifuged and incubated the samples for 10 min at 37 °C. The absorbance of the supernatant was recorded using a spectrophotometer at 535 nm [36,39].

Anti-haemolytic activity: Initiating free radicals are generated by 2,2,-azobis (2-amidinopropane) dihydrochloride (AAPH), which could induce LPO and attack the RBC membrane and eventually cause haemolysis. Blood samples were obtained from a blood bank in heparinized tubes. Firstly, the blood was centrifuged (3000 rpm for 10 min), and the pellets (RBCs) were washed three times with normal saline. A human RBC suspension (5% haematocrit) was prepared in normal saline. The cell suspension was pre-incubated with ascorbic acid and *SA* leaf hydro-alcoholic extract and essential oil in various concentrations (50, 100, 500, 1000, 1500, and 2000 µg/mL) at 37 °C for 1 h and then subjected to a haemolytic activity assay. Then, the treated cells were incubated with AAPH solution (a final concentration of 200 mM) at 37 °C for 3 h and centrifuged (3000 rpm for 10 min). Finally, the degree of haemolysis was assessed using a record of the absorbance at 570 nm. The control group was a reacting mixture without the samples [40,41]. The percentage of anti-haemolysis was calculated using the following equation:

% Inhibition=[(Abs. of control−Abs. of samples)×100]÷Abs. of control

Statistical Analysis: All the above in vitro experiments were performed in triplicate (x = 3). Data were presented as the mean ± SEM. All results were analysed using one-tailed t-tests followed by ANOVA (Graphpad prism 9.0.0). IC_50_ and EC_50_ assays were calculated using Graphpad prism. All in vivo results are expressed as the mean ± SEM. (n = 5) and were analysed using a t-test followed by ANOVA. The levels of significance are presented as * *p* < 0.05, ** *p* < 0.01, *** *p* < 0.001 compared to negative control groups.

## 3. Results

### 3.1. LC-MS Interpretation of Hydro-Alcoholic Extract

**Figure 1:**
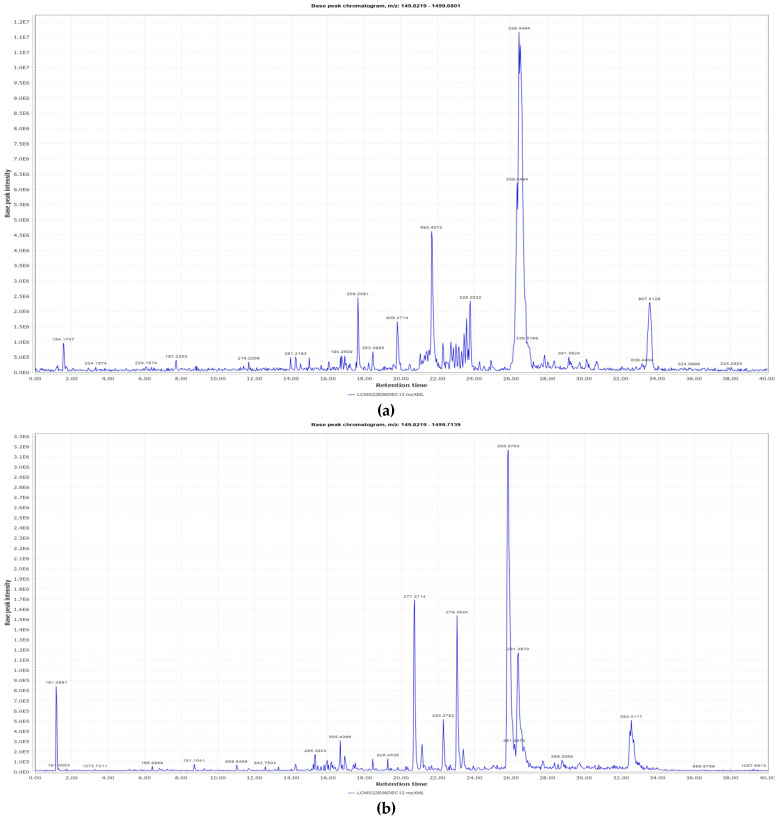
LC-MS of SAE. (**a**): Positive ions of LC-MS of SAE. (**b**): Negative ions of LC-MS of SAE.

The identified active metabolites in SA leaves were obtained using qualitative LC/MS analysis. According to the LC/MS results (Figure 1a,b), the SA hydro-alcoholic extract was reported to contain 40 important constituents (Table 1).

### 3.2. TPC and TFC

TPC and TFC were assessed using gallic acid and quercetin respectively. SAE has a phenolic activity gallic acid equivalent of 86.607 mg per g of dry SAE, as depicted in Figure S1. The flavonoid concentration of SAE was computed to be 333.19 mg of quercetin eq/g dry weight (Figure S1).

### 3.3. In Vitro Antioxidant Activities

**Figure 2:**
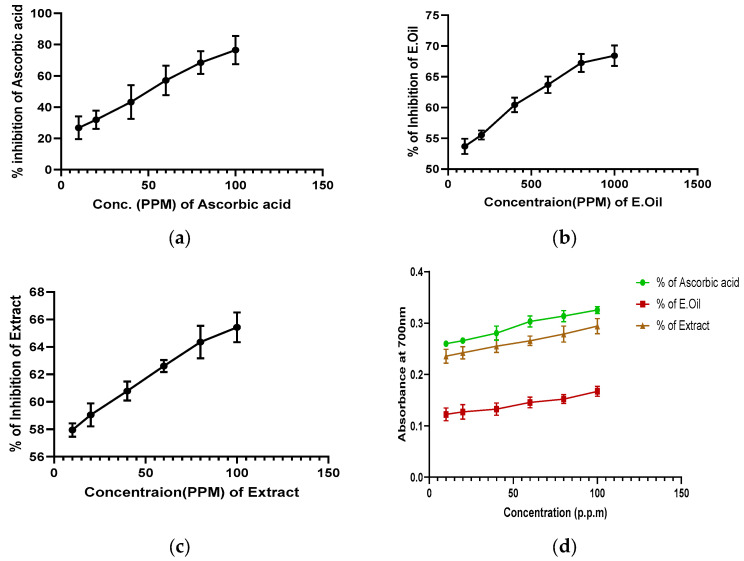

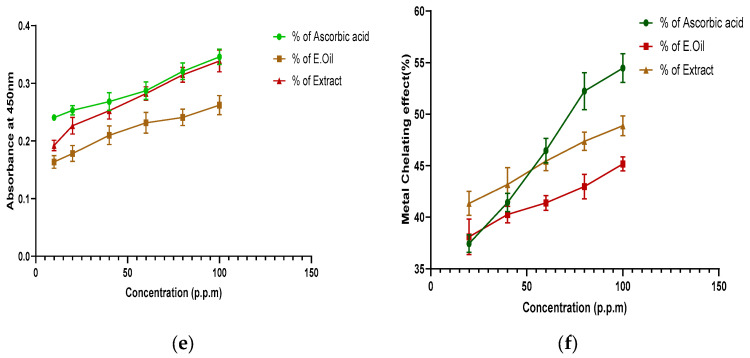
(**a**–**c**): IC_50_ of ascorbic acid, *SAE*, and *SAEO* using DPPH activity. (**d**–**f**): Reducing power, CUPRAC (cupric reducing antioxidant power) and metal chelation assay of ascorbic acid, hydro-alcoholic extract, and essential oil from *SA.* Values are expressed as mean ± SEM (n = 3).

**Figure 3:**
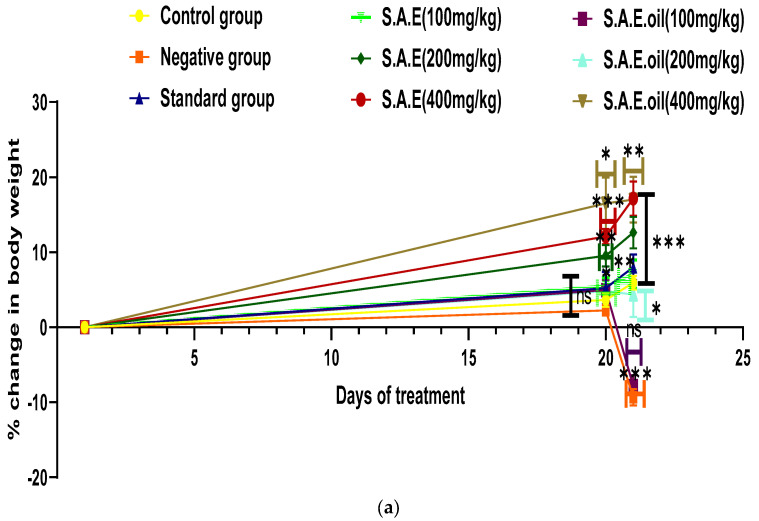

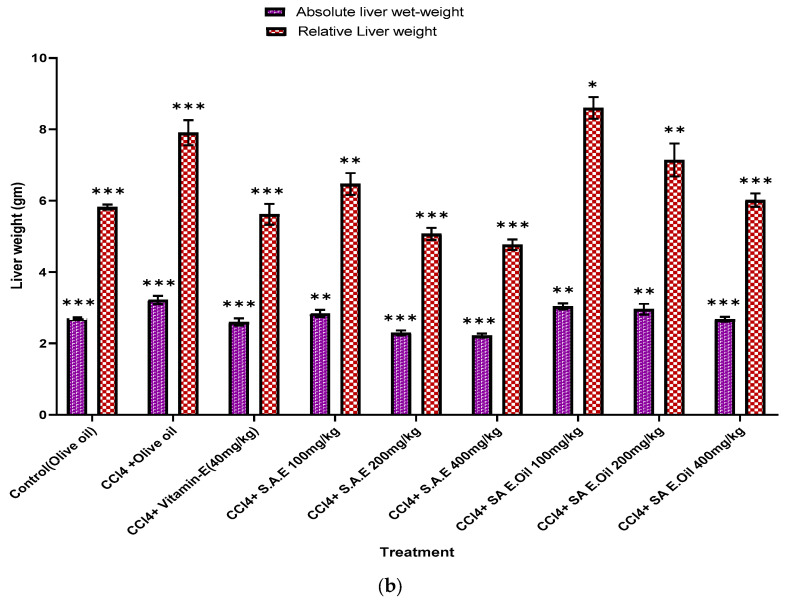
Percent change in body weight and organ index. (**a**) Per cent change in body weight of an animal using carbon tetrachloride, vitamin E, S.A hydro-alcoholic extract, and E. oil (essential oil). (**b**): Organ index for an animal using carbon tetrachloride, vitamin E, S.A hydro-alcoholic extract, and E. oil (essential oil). The levels of significance calculated by unpaired *t* test are presented as * *p* < 0.05, ** *p* < 0.01, *** *p* < 0.001 compared to negative control groups.

The IC_50_ values of SAE and SAEO in DPPH radicals were 1.2 ± 0.2 and 73.4 ± 6.1 μg/mL, respectively (Table 3 and Figure 2a–c). Figure 3a–c shows the sample’s scavenging effects by DPPH radical in the following order SAE > SAEO. 

The ferric ion reducing power of SAE and SAEO (10–100 μg/mL) from *SA* were computed. The ranking order for the reducing potential was ascorbic acid > SAE > SAEO. Significantly higher reducing power was evident in SAE (0.3 ± 0.01 at 100 μg/mL) compared to SAEO (0.17 ± 0.01 at 100 μg/mL), whereas, the standard was shown to have a significant reducing power of 0.33 ± 0.01 at 100 μg/mL (Figure 2d). The cupric ion (Cu^2+^) reducing power of ascorbic acid (0.35 ± 0.01 at 100 μg/mL), SAE (0.34 ± 0.01 at 100 μg/mL) and SAEO (0.26 ± 0.01 at 100 μg/mL) were also calculated (Figure 2e). Significantly higher cupric ion (Cu^2+^) reducing power was evident in SAE compared to ascorbic acid and SAEO.

The hydro-alcoholic extract had chelating activity by reducing the Fe^2+^–ferrozine complex in a dose-dependent manner compared to SAEO (10–100 μg/mL) (Figure 2f). The metal chelating effect of SAE and SAEO had an EC_50_ value at 73.1 ± 3.7 μg/mL, 147.2 ± 20.3 μg/mL, and 362.5 ± 23.5 μg/mL (Table 2). Our study results showed that the metal chelating ability of the samples can be graded as SAE > SAEO. SAE and SAEO exhibited prominent metal chelating activities.

### 3.4. Acute Oral Toxicity

Prior to evaluating the in vivo antioxidant efficacy of the test hydro-alcoholic extract and oil, their acute toxicity was ascertained in accordance with the guidelines set forth by OECD 423. The hydro-alcoholic extract and essential oil were administered orally to three distinct groups of three experimental animals, with each group receiving one of three defined doses (500, 1000, and 2000 mg/kg per os (po)). There was an absence of mortality in animals receiving all doses, and the animals exhibited no indications of abnormal locomotion, seizures, or writhing at a dosage of 2000 mg/kg. This dose was deemed safe and therefore selected as the appropriate dose. No abnormalities or change in the signs and behaviour of the animals were observed for 14 days. Therefore, the hydroalcoholic extract and essential oil preparations were deemed to be safe.

### 3.5. Change in Body Weight and Organ Index

Figure 3 displays data showing a considerable and constant increase in body weight for mice given SAE, SAEO, or vitamin E. On the other hand, the negative control group showed weight loss. The mean standard deviation for weight gain during the study period was 5.51 ± 1.11% for the hydro-alcoholic extract at 100 mg/kg, 9.51 ± 1.44% at 200 mg/kg, and 12.11 ± 1.04% at 400 mg/kg; for essential oil, the corresponding values were 5.01 ± 1.44% at 100 mg/kg, 4.8 ± 2.80% at 200 mg/kg, and 16.52 ± 3.50% at 400 mg/kg. During the 21-day study, animals in the negative control group lost −9.3371 ± 1% of their starting weight, while animals in the positive control group gained 8.0 ± 1.7%. Weight loss was reduced by about 2% when 100 mg/kg of essential oil was administered, while SAE and SAEO at higher doses continued to promote weight gain. In the summary for the negative control group, the absolute change in liver wet weight demonstrates that there was a notable rise in liver wet weight to 3.3 ± 0.09 g as compared to 2.7 ± 0.03 g in the normal control group. In comparison to the SAEO-induced group, the liver wet weight decreased to 2.8 ± 0.1 g, 2.3 ± 0.07 g, and 2.2 ± 0.06 g, and to the SAE-induced group, it decreased to 2.8 ± 0.08 g, 2.7 ± 0.13 g, and 2.6 ± 0.05 g, which were significantly lower. A dose-dependent effect of SAE and SAEO was shown in the relative organ weight of mice. SAE has significant results as compared to SAEO. Vitamin E (5.6 ± 0.29 g), SAE, and SAEO have significantly lower results than negative control (8.2 ± 0.29 g). The relative organ weight of SAE was 6.5 ± 0.31 g, 5.1 ± 0.17 g, and 4.8 ± 0.15 g, whereas the relative organ weight of SAEO was 7.6 ± 0.19 g, 6.4 ± 0.35 g, and 5.8 ± 0.13 g.

### 3.6. In Vivo Hepato-Protective Activity

**Figure 4:**
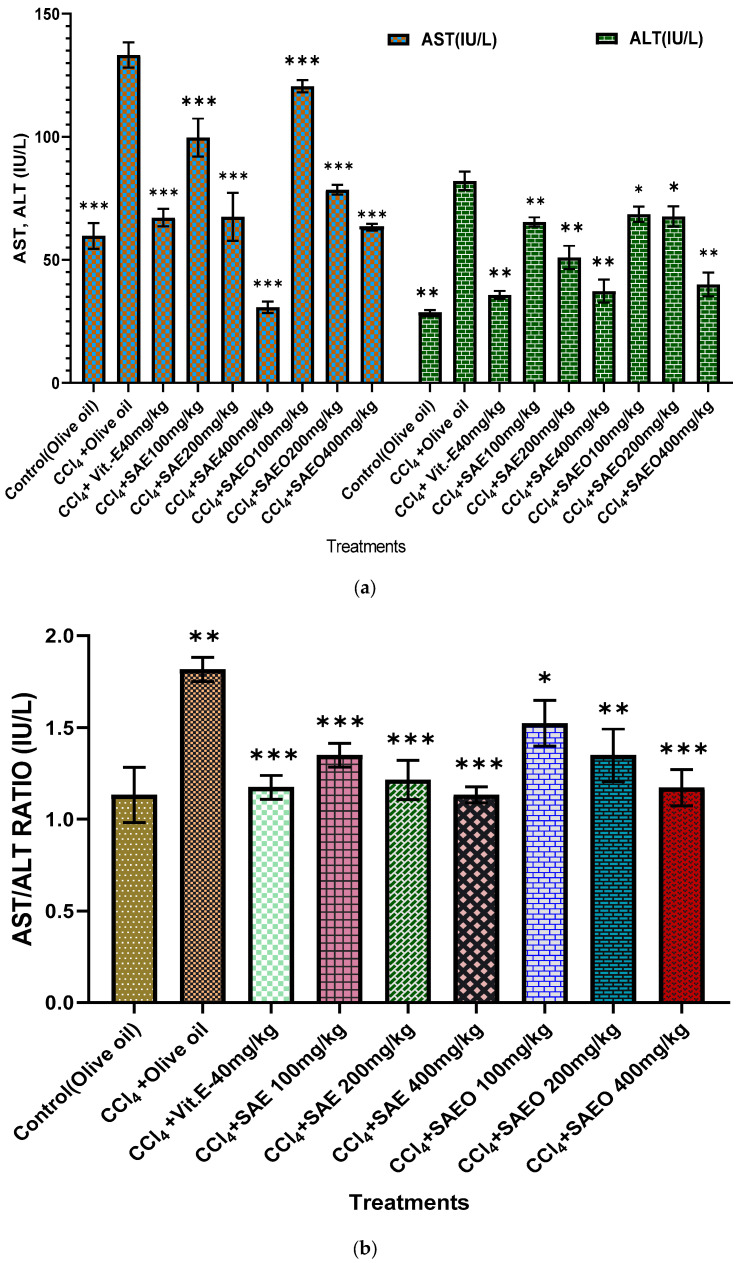
In Vivo Hepato-protective Activity. (**a**,**b**): AST, ALT, and AST/ALT ratio for animals using carbon tetrachloride, vitamin E, S.A hydro-alcoholic extract, and E. oil (essential oil). The levels of significance calculated by unpaired *t* test are presented as * *p* < 0.05, ** *p* < 0.01, *** *p* < 0.001 compared to negative control groups.

Hepatomegaly, serologic alterations, and elevated activity of AST, ALT, and the AST/ALT ratio are all markers of hepatotoxicity, and all were produced with CCl_4_ administration. As shown in Figure 4, the level of all these enzymes and the ratio were considerably (*p* < 0.01) raised with the CCl_4_ treatment and were considerably (*p* < 0.001) alleviated with the post-administration of SAE at 100 mg/kg, 200 mg/kg, and 400 mg/kg orally after CCl_4_. The effectiveness of SAEO increased with increasing doses from 100 mg/kg, 200 mg/kg, and 400 mg/kg. The standard group had considerably lowered hepato-protective levels compared to the negative control group. Since a trend toward negative values may be indicative of regaining health and vitality, a normalization of their appearance may signal a recovery affinity. These findings provide further evidence that SAE and SAEO block the release of liver function enzymes into the bloodstream, hence lowering their concentrations. The hydro-alcoholic extracts’ ability to lower plasma enzyme levels shows that they protect animal livers from CCl_4_ hepatotoxic effects.

### 3.7. In Vivo Nephroprotective Activity

**Figure 5:**
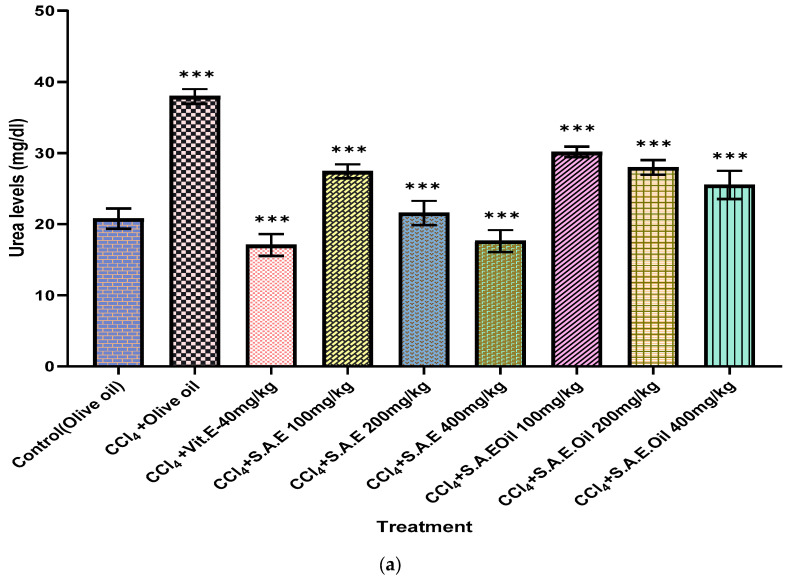

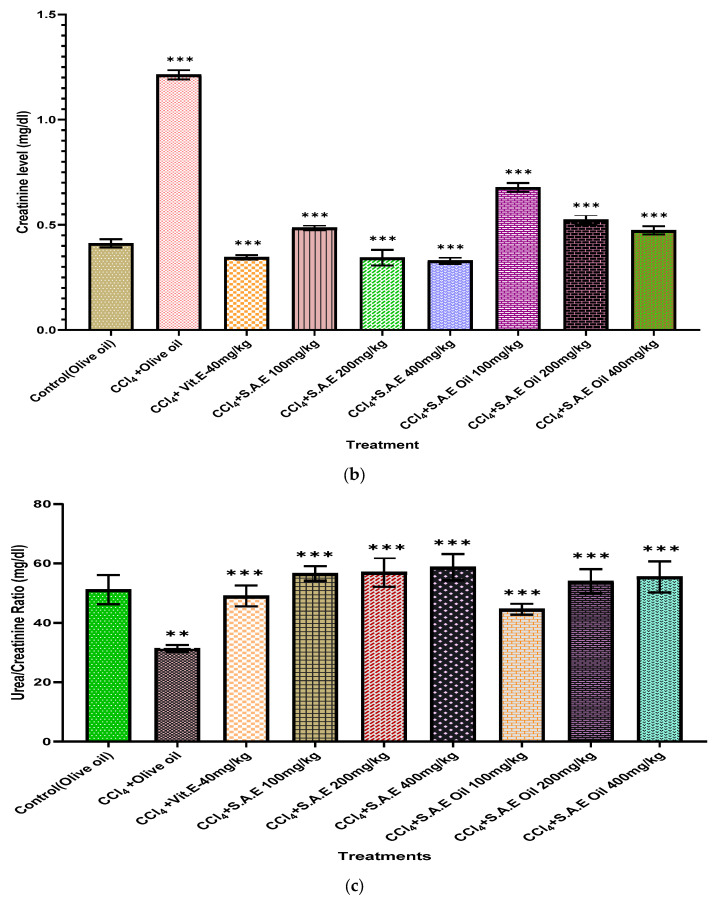
In Vivo Nephroprotective Activity. (**a**–**c**): Urea, creatinine, and the urea/creatinine ratio for animals treated with CCL_4,_ vitamin E, S.A hydro-alcoholic extract, and E. oil (essential oil). The levels of significance calculated by unpaired *t* test are presented as ** *p* < 0.01, *** *p* < 0.001 compared to negative control groups.

Nephrotoxicity was demonstrated by serological alterations in kidney function, including elevated levels of creatinine, urea, and the urea/creatinine ratio, after intraperitoneal delivery of CCL_4_. Treatment with CCl_4_ considerably (*p* < 0.01) increased serum urea, creatinine, and the urea/creatinine ratio, as summarized in Figure 5. However, oral delivery of SAE (100 mg/kg, 200 mg/kg, and 400 mg/kg) after CCl_4_ dramatically (*p* < 0.001) lower these functional indicators toward near-normal values. The effectiveness of SAEO increased with increasing doses of 100 mg/kg, 200 mg/kg, and 400 mg/kg. The levels of these parameters were higher in the negative control group of mice as compared to the normal control animals. Upon receiving routine medical care, these values may return to normal, which would suggest a healing affinity and a consequent shift toward positive values, signifying recovery. These findings show that SAE and SAEO are effective in lowering blood levels by preventing kidney function abnormality. The hydro-alcoholic extracts’ ability to lower plasma enzyme levels suggests that they protect animal kidneys from CCL_4_ nephrotoxic effects.

### 3.8. In Vivo Antioxidant Activity

**Figure 6:**
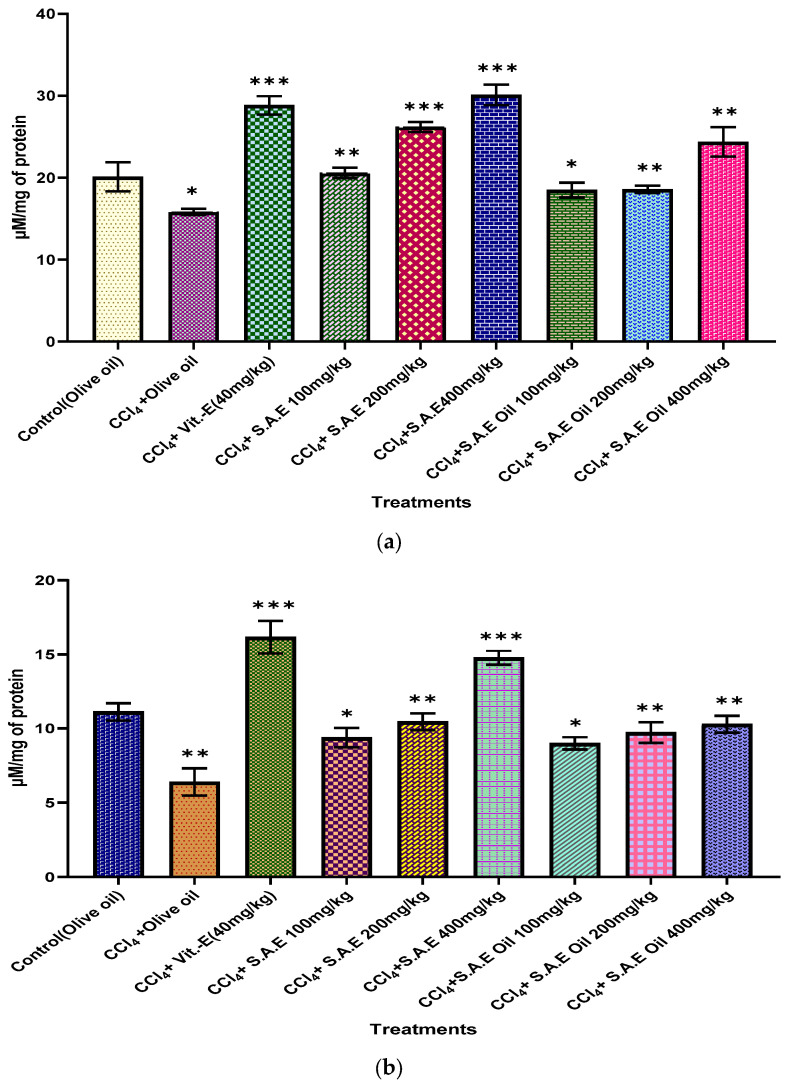

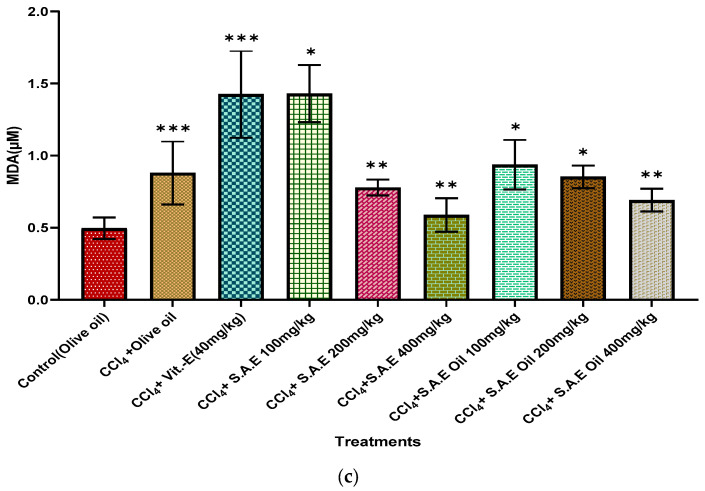
In Vivo Antioxidant Activity. Catalase (**a**), reduced glutathione (**b**) and lipid peroxidation (**c**) activity in animals treated with carbon tetrachloride, vitamin E, S.A hydro-alcoholic extract and E. oil (essential oil). The levels of significance calculated by unpaired *t* test are presented as * *p* < 0.05, ** *p* < 0.01, *** *p* < 0.001 compared to negative control groups.

Fatty acid build-up caused by CCL_4_ administration increases ROS generation in liver tissues. We measured glutathione reductase (GR), catalase (CAT), and lipid peroxide (LPO) in CCL_4_-induced mouse liver tissues to learn more about the antioxidant impact of SAE and SAEO (Figure 6a–c). The enzyme activity was drastically decreased with CCl_4_. SAE and SAEO therapy (100, 200, and 400 mg/kg) up-regulates the activity of these enzymes, bringing them close to that in the normal control group. According to the findings, SAE is superior to SAEO in its ability to reduce oxidative stress in hepatocytes by boosting the activity of antioxidant enzymes. When SAE was given to CCL_4_-induced mice, the CAT activity in the homogenate was significantly (*p* < 0.05) higher in the treated groups (IV, V, and VI) than in the untreated group (II). Similar increases (*p* < 0.05) in CAT activity in the homogenate were observed after SAEO treatment. The tissue CAT activity was greatest (*p* < 0.05) for the 400 mg/kg hydro-alcoholic extract group compared to the other preparations. Figure 6c shows that compared to the normal group, CCl_4_ administration led to a statistically major (*p* < 0.05) rise in serum LPO generation, while SAE and SAEO administration reduced the amount to nearly that in the control group. The outcome of the leaf hydro-alcoholic extract on liver tissue was dose-dependent and greatest at 400 mg/kg and considerably (*p* < 0.05) better than the 400 mg/kg dose of SAEO. Indications are promising that SA hydro-alcoholic extract treatment can shield against free radical damage by decreasing the rate at which lipids are oxidized.

### 3.9. Anti-Haemolytic Activity of SAE and SAEO

**Figure 7:**
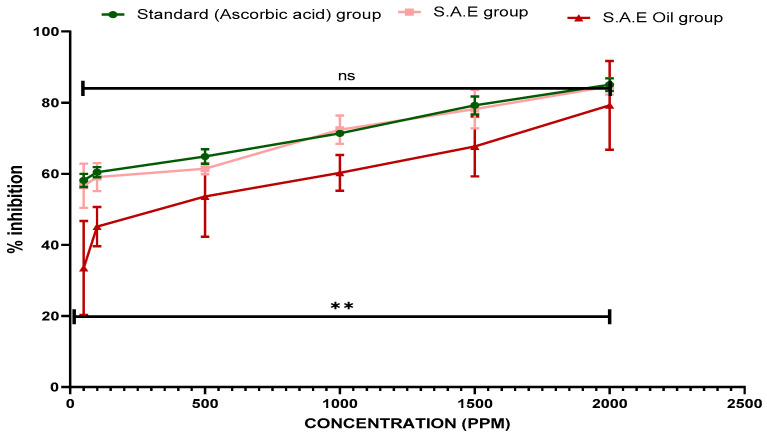
Haemolytic activity with ascorbic acid, S.A hydro-alcoholic extract and E. oil (essential oil). The levels of significance calculated by unpaired *t* test are presented as ** *p* < 0.01 compared to negative control groups.

The results showed that the erythrocyte membrane lysis was prevented using concentrations of SAE and SAEO ranging from 100 to 2000 µg/mL. With an increase in concentration, the inhibitory effect of ascorbic acid, SAE, and SAEO on haemolysis peaked at 85.1 ± 1.8%, 84.6 ± 2.3% and 79.2 ± 1.8% at 2000 µg/mL. Ascorbic acid was used to make comparisons of the results. Lysis of lysosomes occurs during inflammation, and their contents are identical to those in red blood cell membranes. Haemolysis and haemoglobin oxidation are both outcomes of hypotonic stress on red blood cells. Figure 7 displays the results of an experiment in which the haemolytic activity of SAE and SAEO was tested on normal human erythrocytes. The IC_50_ values for SAE and SAEO for inhibiting haemolysis were 30.2 ± 0.3 µg/mL and 232.2 ± 0.4 µg/mL, respectively; for comparison, the IC_50_ value for ascorbic acid was 23.08 ± 0.3 µg/mL (Table 4). When compared to SAEO, SAE’s anti-haemolytic action is superior.

### 3.10. Histopathology of Liver for Hepatoprotective Activity

**Figure 8:**
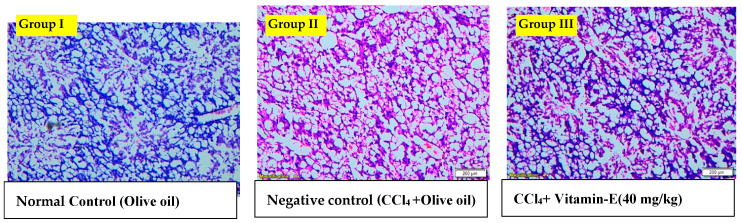

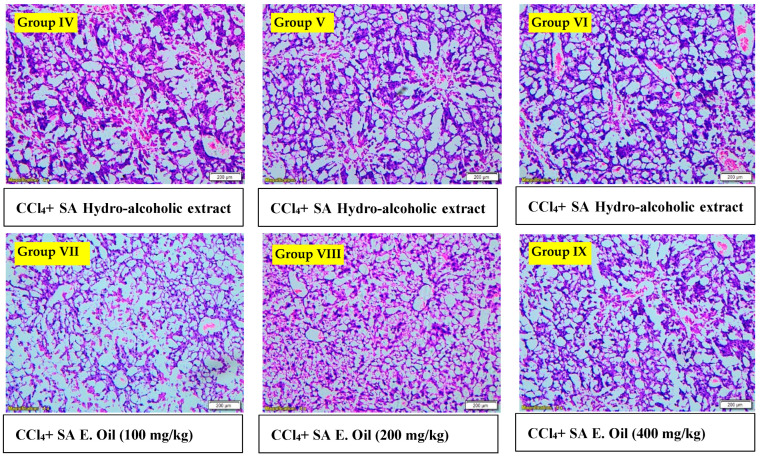
Histopathological analysis of CCl_4_-induced toxicity in mice.

A histopathological analysis of CCl_4_-induced toxicity in mice was used to examine the hepatoprotective and curative effects of SAE and SAEO. The hepatic sections of the normal group showed typical hepatocyte architecture and distinct sinusoids (Figure 8). On the other hand, liver sections from CCl_4_-treated mice, show a wide variety of histological changes. These include altered hepatocyte morphology, plasmolysis, nuclear enlargement, connective tissue infiltration with prominent necrosis, blocking of the central vein, and infiltration of neutrophils. Furthermore, hepatocytes, cell membranes, and the central vein were all in good working order in the livers of vehicle-treated control animals. Our study showed significant dose-dependent recovery of SAE-treated mice that had previously been given CCL_4_ at 100 mg/kg, 200 mg/kg, or 400 mg/kg, demonstrated in the liver’s histological indications, with smaller and less severe inflammatory cell infiltration and less congestion when compared to the CCl_4_-treated group. The impact of SAEO was less than that of SAE. Some degenerative changes were seen in vitamin E-treated animals following CCL_4_, but only in contrast with the normal group.

## 4. Discussion

Oxidative stress is a process that occurs due to unevenness between production, accumulation, and detoxification of ROS in cells and tissues. A decrease in ATP production (mitochondria) is associated with the amplification in ROS generation and oxidative stress that can cause cellular dysfunction. ROS are involved in many biotic processes including cell growth, differentiation or multiplication, and death [42].

Two mechanisms are involved in ROS causing hypertension. Firstly, increased oxidative stress in animals causes amplified sympathetic and declined parasympathetic nerves in the heart, and also amplified plasma lipo-peroxidation levels that cause an increase in arterial pressure [43]. Secondly, exacerbated ROS production leads to a reaction between the superoxide (O_2_^−^) anions and NO present in our body to form peroxy-nitrite, which causes a reduction in NO levels and diminishes NO-related smooth muscle relaxation [44]. The authors of a previous study confirmed that oral vitamin C treatment can decrease the oxidative profile by lowering blood pressure and sympathetic modulation [43]. Oxidative stress can also promote the propagation of vascular smooth muscle and chronic pressure overload exerted on the left ventricle, which plays a significant pathological role in vascular and cardiac alteration in hypertension [45]. According to the previous authors, intake of an antioxidant-rich diet can decrease the threat of hypertension. It was also proved that ethanolic extract from *Cissus quadrangularis* promoted eNOS and inhibited ROS production and inflammatory cytokines that lead to improved endothelium-dependent relaxation in hypertensive rats [46].

Imbalance in ATP synthesis (declined) and oxidative stress (increased) related to mitochondria can cause a deficiency in Pvalb neurons that help in the distortion of neuropsychiatric disorders such as bipolar disorders (BDs), obsessive-compulsive disorder (OCD), major depression, and schizophrenia [47,48]. Elevated ROS cause the amplification of oxidative stress that leads to an increase in the aggressiveness of negative symptoms in schizophrenic patients [42]. Researchers suggested that curcumin improves SCZ (schizophrenic)-like behavioural alterations after measuring oxidative stress indicators occur in animals [49]. According to the researchers, oxidative stress lowered the glutathione peroxidase, CAT, and GR activities in PD (Parkinson’s disorder) patients. A decreased GSSG ratio indicates a key role in the apoptosis of substantia nigra in PD patients [50]. A significant role of ROS was reported in the development of PD in both pre-clinical and clinical studies [51].

An antioxidant can inhibit oxidative damage and its capability to entrap the free radicals via various mechanisms such as scavenging and chelating for the free radical that inhibits lipid oxidation. The adverse ability of free radicals is mitigated with antioxidants, which protect cells from damage. The antioxidative phytochemicals present in vegetables, grains, and fruits have a great contribution to the inhibition of human disease as well as the enhancement of food quality [52]. Plants are very noble antioxidant sources and have been used as medicine since early times. Natural sources of antioxidants have attracted researchers’ interest because they are inexpensive and natural [53].

Since ancient times, various medicinal plants (more than 80,000 species) have been conventionally used as medicines in numerous native medication systems for the treatment of different conditions. However, only 25% of species have been used as prescribed remedial products [54,55]. The Indian Himalayan range is the richest biodiversity hotspot and has one of the broadest varieties of plant species on the globe [56]. A number of studies described the occurrence of numerous phenolic compounds in Himalayan plants [57]. *S. anquetilia* (Rutaceae) is a perennial, erect, ornamental shrub in the Western Himalayas. Conventionally, *SA* leaves have been used for the treatment of various diseases such as headache, smallpox, fever, paralysis, pneumonia, and cancer, as an insect (especially snake and scorpion) poison, and as an anti-inflammatory and anti-diabetic agent. Hence, our study was designed to accomplish phytochemical testing such as the LC-MS techniques of a hydro-alcoholic extract for the identification of a number of bioactive constituents in SA leaves, which has shown antioxidant and anti-haemolytic activity against CCL_4_ intoxication [58]. Overall, flavonoids and amino acids were the most predominant constituent found in SAE. Moreover, purines increased to the third level. On the other hand, alkaloids, peptides, and carboxylic acids were present in the lowest structures, respectively (Table 1).

It has been suggested that polar molecules (such as polyphenolic substances) present in plant hydro-alcoholic extracts contribute to increasing antiradical activity. Phenolic compounds seem to be good candidates for their antioxidant activities because they have the ability to trap free radicals and, consequently, delay the auto-oxidation of lipids [59]. SAE has free radical scavenging activity, and the DPPH model demonstrates that it is most effective at lower concentrations. Though, the antioxidant potential of SAE was found to have higher efficacy compared to the standard drug ascorbic acid. Our study revealed that SAE has significant antioxidant activity due to the presence of TFC compared to SAEO. In addition to antioxidant activity, flavonoids have the ability to stabilize the scavenging chemicals in ROS flooding [60]. In addition to flavonoids, other secondary metabolites such as polyphenols, amino acids, and alkaloids were also reported to have strong antioxidant activity [61]. There was a significant difference between the IC_50_ values for DPPH free radical scavenging activities between SAE and SAEO. SAE and SAEO have the capacity to guard against the detrimental effects of free radicals in the biological system, as evidenced by their reduction and free radical scavenging actions [59].

Antioxidant activity has also been evaluated using other methods such as metal iron chelation, metal ion (FRAP), and copper reduction (CUPRAC), which represent a significant indicator of the antioxidant power of hydro-alcoholic extract and essential oil from SA. The reducing power of SA plant hydro-alcoholic extracts and oil is dose-dependent (concentration-dependent). This is probably due to the presence of hydroxyl groups in phenolic compounds that can be used as electron donors. Therefore, antioxidants in SA are considered to reduce and inactivate oxidants [58].

CCL_4_ intoxication can cause a decrease in body weight that relates to liver damage [62]. In the current study, mice in the control group showed an increase in body weight, whereas the other negative control group showed a significant decrease in body weight on the day of sacrifice (24 h after CCl_4_ treatment). These results are reliable evidence that SAE exerted significant inhibitory action on the CCl_4_-induced changes in body weight compared to SAEO. Other authors found that marked changes occur in nodulation and enlargement of the liver when treated with CCl_4_, and these changes were related to an increase in liver weights [61]. However, test substance-treated mice showed a decrease in hepatic enlargements and nodule formations compared to the CCl_4_ group resulting in a significant decrease in liver weights. The findings in our study have clear evidence that SAE induced favourable hepatoprotective effects on CCl_4_-induced acute liver injury in mice compared to SAEO.

CCL_4_ has been used to induce liver and kidney damage in experimental animals. CCL_4_ induced liver and kidney toxicity by elevating the levels of AST, ALT, urea, and creatinine, while different doses of *SA* hydro-alcoholic extract and essential oil decreased the levels of AST, ALT, urea, and creatinine compared to the negative group, similar to a previous study [63]. Several studies have reported that CCl_4_ increased oxidative stress, which led to a rise in LPO of polyunsaturated fatty acids (MDA level) and a decrease in GR and CAT levels, and this led to hepatotoxic issues such as fatty liver cirrhosis, fibrosis and carcinogenicity [64]. In the current study, treatment of mice with SAE and SAEO significantly decreased the MDA level and increased GR and CAT levels after CCl_4_-induced oxidative stress. This also agrees with our studies indicating that SAE and SAEO show significant progress in CAT activities in liver tissues [65]. Peroxidation of the lipid membrane interrupts the permeability of various organelles (endoplasmic reticulum and mitochondria) and plasma membranes which can cause the loss of calcium cell detention and homeostasis leading to leakage of microsomal enzymes and cell damage [66]. The liver homogenate from the SAE- and SAEO-treated mice were displayed at a considerably lower level than the LPO level in cells. This is a strong indication that *SA* hydro-alcoholic extract and essential oil can exhibit an up-regulation of the antioxidant defence mechanisms in the tissues of experimental animals [65].

Histopathological examination of the mouse liver and spleen proved our biochemical and molecular results and showed various modifications, such as severe deterioration and necrosis of hepatocytes and fatty alterations, and showed the presence of inflammatory cells. That is why our data indicated that SAE and SAEO improved serum AST, ALT, and the AST/ALT ratio levels that were elevated after CCl_4_ intoxication [67]. This may be associated with liver damage and failure to metabolize lipids by liver cells. Furthermore, CCl_4_ administration led to significant rises in creatinine, urea, and urea/creatinine levels, as compared to the normal control group, which indicates that CCl_4_ induced nephrotoxicity. Our results proved that the CCL_4_ intoxication was treated by SAE and SAEO [63].

Due to the presence of high polyunsaturated fatty acids in their membrane, erythrocytes are highly sensitive to oxidative stress and act as the first target of free radical attack. Therefore, erythrocytes are often used to assess the in vitro anti-haemolytic and antioxidant potential of different plant compounds [68]. SAE and SAEO have shown significant antioxidant activity by stabilizing the free radicals and increasing erythrocyte oxidative stress resistance. Moreover, high phenolic components in hydroalcoholic extracts could donate more than one electron to nullify the AAPH radical while inhibiting haemolysis as compared to SAEO [69].

## 5. Conclusions

In conclusion, *SA* has a wide range of phenolic compounds (flavonoids) that exhibit potent antioxidant and antiradical properties. When compared to the reference substance (ascorbic acid), *SA* demonstrated impressive DPPH scavenging capabilities. Significant reduction capability of *SA* in FRAP and CUPRAC and positive metal chelating assays were also observed. *SA* has been shown to have protective effects against CCl_4_-induced hepatotoxicity and nephrotoxicity, in addition to its antioxidant properties, similar to that of vitamin E, which may be attributable to the presence of flavonoids. Our results show that *SA* leaf hydro-alcoholic extract and essential oil effectively attenuate AAPH-induced haemolysis on human RBCs. Its potential antioxidant properties make it a promising treatment for haemolytic anaemia, suggesting it could be used in the food and pharmaceutical industries. These results prove that the hepatoprotective and nephroprotective activity of SA could be due to its strong antioxidant properties. We recommend further detailed studies to elaborate on the cellular and molecular mechanisms of these antioxidant properties.

## Figures and Tables

**Table 1:** Chemical Analysis of *SA* Hydro-alcoholic Extract.

R.Time	Compound Name	Category	Ion	Formula	Structure	Exact Mass	Observed Mass
1–55	Suberic Acid	Dicarboxylic Acid	Positive	C8H14O4	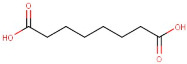	174.089	174.1747
6–09	Methyl Jasmonate	Jasmonate Ester	Positive	C13H20O3	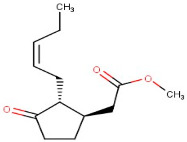	224.141	224.1574
6–36	L-Carnosine	Peptide	[M+H]+	C9H14N4O3	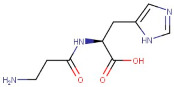	226.23	224.1574
7–28	3-Hydroxy-DL-Kynurenine	Amino Acid	[M+H]+	C10H12N2O4	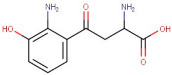	224.21	224.0224
7–72	3,4-Dihydroxy-L-Phenylalanine	Amino Acid	[M+H]+	C9H11NO4	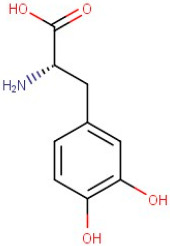	197.19	197.2303
13–96	1,10-Phenanthroline Monohydrate	*Hetero-cyclic organic Compound*	Positive	C12H8N2	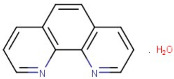	180.068	181.2362
14–24	Acacetin	Flavonoids	Positive	C16H12O5	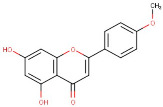	284.068	281.2183
14–51	Linoleic Acid	Omega-6 Fatty Acids	Positive	C18H32O2	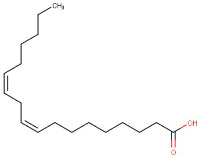	280.24	279.1261
14–99	Leucylleucyltyrosine	Peptide	Positive	C21H33N3O5	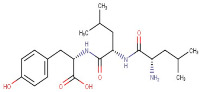	407.242	403.2747
16–04	Butenyl Glucosinolate/Gluconapin	Amino Acid	Positive	C11H19NO9S2	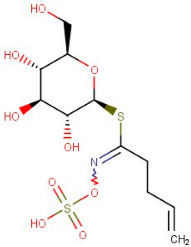	373.05	373.1734
16–93	Sinapine	Alkaloid	Positive	C16H24NO5	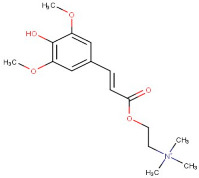	310.165	313.3769
17–65	Chalcone	Flavonoid	Positive	C15H12O	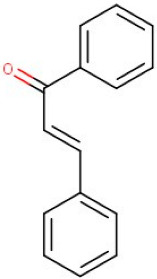	208.088	209.2091
21–53	Adenosine-5′-Diphospho-Glucose Disodium Salt	Purine	[M+H]+	C16H25N5O15P2	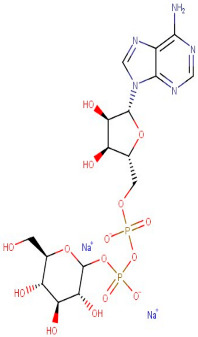	589.32	634.6143
22–28	Isorhamnetin-3-O-Rutinoside	Flavonoid	Positive	C28H32O16	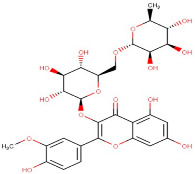	624.169	625.5021
23.00	2′-Deoxyadenosine-5′-Diphosphate Sodium Salt	Purine	Positive	C55H72MgN4O5	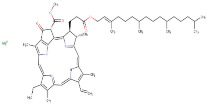	868.55	556.5203
23–78	Scoulerine	Alkaloid	Positive	C19H21NO4	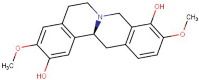	327.147	329.2032
26–34	6-(Gamma,Gamma-Dimethylallylamino)Purine Riboside	Purine	Positive	C15H21N5O4	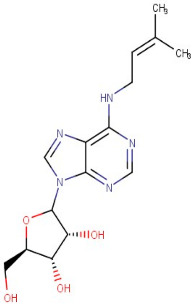	335.159	338.4494
26–89	5-Aminoimidazole-4-Carboxamide-1-Beta-D-Ribofuranosyl 5′-Monophosphate	Purine	Positive	C9H15N4O8P	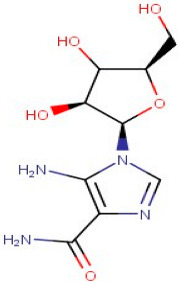	338.062	338.4156
3–57	Esculin Sesquihydrate	Carbohydrate	Positive	C15H16O9	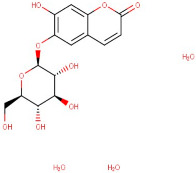	340.079	338.5169
	Guanosine-5′-Diphosphate-D-Mannose Sodium Salt	Carbohydrate	[M+H]+	C16H25N5O16P2	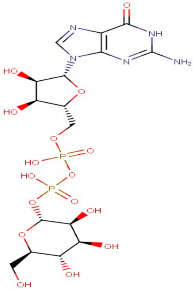	605.34	607.4128
1–16	D-(-)-Quinic Acid	*Monocarboxylic Acid*	Negative	C7H12O6	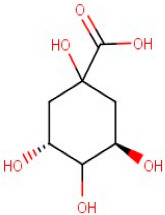	192.063	191.0891
15–31	Pentachlorophenol	Organo-Chlorine Compound	[M-H]+	C6HCl5O	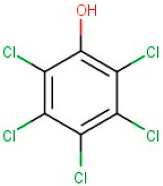	266.34	265.3923
18–45	Piperacillin Sodium Salt	Ureidopenicillin	Negative	C23H27N5O7S	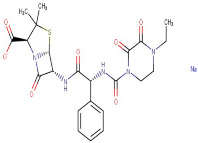	517.163	513.5256
19–27	Petunidin-3-O-(6″-O-(4‴-O-E-Coum)-Alpha-Rhamnopyranosyl-Beta-Glucopyranosyl)-5-O-Beta-Glucopyranoside/	Flavonoid	[M-2H]+	C43H49O23	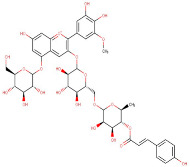	933.84	926.4535
20–73	6-Phosphogluconic Acid Barium Salt Hydrate	Carbohydrate	Negative	C6H13O10P	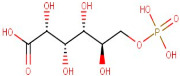	276.024	277.3714
21–14	L-Carnosine	Peptide	Negative	C9H14N4O3	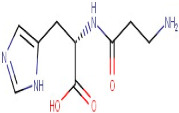	226.106	227.2280
22–30	D-Glucosamine-6-Phosphate Sodium Salt	Amino Sugar	Negative	C6H14NO8P	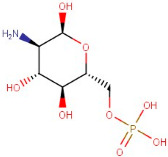	259.045	253.2782
25–88	2′-Deoxyinosine	Purine	Negative	C10H12N4O4	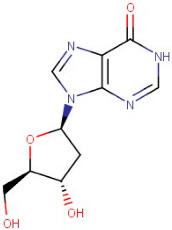	252.085	255.3703
26–19	Luteolin	Flavonoid	Negative	C15H10O6	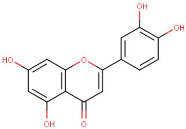	286.047	281.3870
28–78	Naringenin	Flavonoid	Negative	C15H12O5	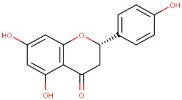	272.068	269.3066
32–57	Linarin	Flavonoid	Negative	C28H32O14	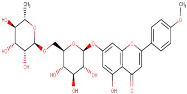	592.179	283.4117
32–70	Xanthosine	Purine	Negative	C10H12N4O6	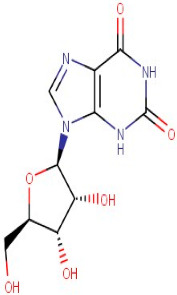	284.075	283.4454
33–42	Acacetin	Flavonoid	Negative	C16H12O5	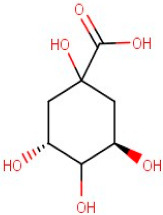	284.068	283.4117

**Table 2:** Important Chemical Constituents of *SA* Essential Oil.

Calculated KI	Chemical Name	Mass (g/mol)	Category	Chemical Formula	Structure
931	α-Pinene	136.23	Terpene	C_10_H_16_	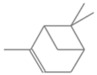
1000	α-Phellandrene	136.23	Cyclic Mono-terpenes	C_10_H_16_	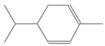
1025.31	γ-Terpinene	136.23	Cyclic Mono-terpenes	C_10_H_16_	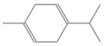
988.61	β-Myrcene	136.23	Aliphatic Mono-terpenes	C_10_H_16_	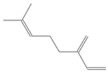
988.73	β-Pinene	136.23	Terpene	C_10_H_16_	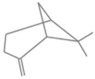
1026.51	Eucalyptol	154.25	Mono-terpenoid	C_10_H_18_O	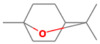
1530.72	α-Selinene	204.35	Sesquiterpenes	C_15_H_24_	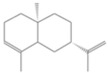
970.78	Cyclohexane, 1-methylene-4-(1-methylethenyl)-	136.23	Cyclic Mono-terpenes	C_10_H_16_	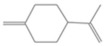
1025.31	β-Phellandrene	136.23	Cyclic Mono-terpenes	C_10_H_16_	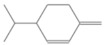
1079.70	Cyclohexene, 1-methyl-4-(1-methylethylidene)-/p-Menth-4(8)-ene	138.25	Mono-terpenoid	C_10_H_18_	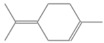
1123.75	2,4,6-Octatriene, 2,6-dimethyl-	136.23	Acyclic Mono-terpenes	C_10_H_16_	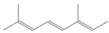
931.07	Bicyclo [3.1.0]hex-2-ene, 2-methyl-5-(1-methylethyl)-/ α-Thujene	136.23	Cyclic Mono-terpenes	C_10_H_16_	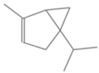
1492.35	Cyclohexane, 1-ethenyl-1-methyl-2-(1-methylethenyl)-4-(1-methylethylidene)-	204.35	Sesquiterpenes	C_15_H_24_	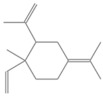
1042.08	1,3,6-Octatriene, 3,7-dimethyl-, (Z)-/Cis-beta-Ocimene	136.23	Acyclic Mono-terpenes	C_10_H_16_	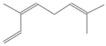
970.90	Bicyclo [3.1.0]hexane, 4-methylene-1-(1-methylethyl)-	194.27	Bicyclic monoterpene	C_12_H_18_O_2_	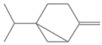
1042.08	trans-β-Ocimene	136.23	Acyclic Mono-terpenes	C_10_H_16_	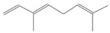
1127.93	Geijerene	162.27	Sesquiterpene	C_12_H_18_	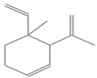
1000.19	3-Carene	136.23	Bicyclic monoterpene	C_10_H_16_	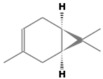
1042.08	β-Ocimene	136.23	Acyclic Mono-terpenes	C_10_H_16_	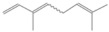
1492.35	Bicyclogermacrene	204.35	Sesquiterpene	C_15_H_24_	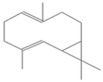

**Table 3:** In vitro antioxidant activity of SA essential oil and hydro-alcoholic extract.

Treatment	IC_50_ of DPPH Assay (µg/mL)	EC_50_ of Metal Chelating Assay (µg/mL)	Reducing Power Assay	CUPRAC Assay
Ascorbic acid	10.4 ± 1.9 ^a^	73.1 ± 3.7	0.33 ± 0.01 ^a^	0.35 ± 0.01 ^b^
*SAEO*	73.38 ± 6.1 *** ^a^	362.5 ± 23.5 *** ^a^	0.17 ± 0.01 *** ^a^	0.26 ± 0.01 *** ^b^
*SAE*	1.2 ± 0.2 *** ^a^	147.2 ± 20.3 *** ^a^	0.30 ± 0.01 *** ^a^	0.34 ± 0.01 *** ^b^

In vitro antioxidant assay of ascorbic acid, *SAE*, and *SAEO.* The levels of significance calculated by unpaired *t* test are presented as *** *p* < 0.001 compared to negative control groups. Analysis of results by One Way ANOVA and level of significance are presented as ^a^: *p* < 0.05, ^b^: *p* < 0.01.

**Table 4:** Anti-haemolytic activity of SA essential oil and hydro-alcoholic extract.

Groups	IC_50_ (µg/mL)
Standard (Ascorbic acid)	23.08 ± 0.3
S.A.E	30.20 ± 0.3 **
S.A.E Oil	232.2 ± 0.4 **

** *p* < 0.01.

## Data Availability

All data generated in this study are provided in the manuscript. No extra data are available.

## Supplementary Materials

The following supporting information can be downloaded at: https://www.mdpi.com/article/10.3390/antiox12061167/s1, Figure S1: A and B showed TPC and TFC of Gallic acid and quercetin. SEM (n = 3).

## Abbreviation

LC-MS	liquid chromatography-mass spectrometry
CCl_4_	carbon tetrachloride
ALT	alanine transaminase
AST	aspartate aminotransferase
SAE	Skimmia anquetilia hydro-alcoholic extract
SAEO	*Skimmia anquetilia* essential oil
TCA	tri-chloro acetic acid
OECD	Organization for Economic Cooperation and Development
LD_50_	lethal dose
p.o	per oral
SGOT	serum glutamic-oxaloacetic transaminase
SGPT	serum glutamic pyruvic transaminase
EDTA	etheylenediaminetetraacetic acid
AAPH	2,2’-Azobis(2-amidinopropane) dihydrochloride
ROS	reacting oxygen species

## References

[1] Rajakumari R., Volova T., Oluwafemi O.S., Rajesh K.S., Thomas S., Kalarikkal N. (2020). Grape seed hydro-alcoholic extract-soluplus dispersion and its antioxidant activity. Drug Dev. Ind. Pharm..

[2] Lee H.G., Kim H.S., Oh J.Y., Lee D.S., Yang H.W., Kang M.C., Kim E.A., Kang N., Kim J., Heo S.J. (2021). Potential Antioxidant Properties of Enzymatic Hydrolysates from *Stichopus japonicus* against Hydrogen Peroxide-Induced Oxidative Stress. Antioxidants.

[3] Munteanu I.G., Apetrei C. (2021). Analytical Methods Used in Determining Antioxidant Activity: A Review. Int. J. Mol. Sci..

[4] Pavithra K.S., Vadivukkarasi S. (2015). Evaluation of free radical scavenging activity of various hydro-alcoholic hydro-alcoholic extracts of leaves from *Kedrostis foetidissima* (Jacq.) Cogn. Food Sci. Hum. Wellness.

[5] Yachamaneni J., Dhanraj S. (2017). Anti-Hepatotoxic and Antioxidant Activity of *Limnanthemum indicum* Against Carbon Tetrachloride Induced Liver Toxicity in Rats. Indian J. Pharm. Educ. Res..

[6] Khan R.A., Khan M.R., Sahreen S. (2012). CCl_4_-induced hepatotoxicity: Protective effect of rutin on p53, CYP2E1 and the antioxidative status in rat. BMC Complement. Altern. Med..

[7] Apak R., Gu K., Mustafa O., Celik S.E. (2008). Mechanism of Antioxidant Capacity Assays and the CUPRAC (Cupric Ion Reducing Antioxidant Capacity) Assay. Microchim. Acta.

[8] Gupta A.K., Chitme H., Dass S.K., Misra N. (2006). Antioxidant activity of Chamomile recutita capitula methanolic extracts against CCl_4_-induced liver injury in rats. J. Pharmacol. Toxicol..

[9] Sravan Kumar S., Singh Chauhan A., Giridhar P. (2020). Nanoliposomal encapsulation mediated enhancement of betalain stability: Characterisation, storage stability and antioxidant activity of *Basella rubra* L. fruits for its applications in vegan gummy candies. Food Chem..

[10] Wintola O.A., Olajuyigbe A.A., Afolayan A.J., Coopoosamy R.M., Olajuyigbe O.O. (2021). Chemical composition, antioxidant activities and antibacterial activities of essential oil from *Erythrina caffra* Thunb. growing in South Africa. Heliyon.

[11] Ganie S.A., Haq E., Hamid A., Qurishi Y., Mahmood Z., Zargar B.A., Masood A., Zargar M.A. (2011). Carbon tetrachloride induced kidney and lung tissue damages and antioxidant activities of the aqueous rhizome hydro-alcoholic extract of *Podophyllum hexandrum*. BMC Complement. Altern. Med..

[12] Torres-Martínez R., García-Rodríguez Y.M., Ríos-Chávez P., Saavedra-Molina A., López-Meza J.E., Ochoa-Zarzosa A., Garciglia R.S. (2018). Antioxidant Activity of the Essential Oil and its Major Terpenes of *Satureja macrostema* (Moc. and Sessé ex Benth.) Briq. Pharmacogn. Mag..

[13] Manca M.L., Lai F., Pireddu R., Valenti D., Schlich M., Pini E., Ailuno G., Fadda A.M., Sinico C. (2020). Impact of nanosizing on dermal delivery and antioxidant activity of quercetin nanocrystals. J. Drug Deliv. Sci. Technol..

[14] Burli S., Chitme H.R., Vrunda K., Jamadar M.J. (2016). Antiproliferative and antioxidant activity of leaves extracts of Moringa oleifera. Int. J. Curr. Pharm. Res..

[15] Ling J.K.U., Chan Y.S., Nandong J., Chin S.F., Ho B.K. (2020). Formulation of choline chloride/ascorbic acid natural deep eutectic solvent: Characterization, solubilization capacity and antioxidant property. LWT-Food Sci. Technol..

[16] Maqsoudlou A., Assadpour E., Mohebodini H., Jafari S.M. (2020). Improving the efficiency of natural antioxidant compounds via different nanocarriers. Adv. Colloid Interface Sci..

[17] Gondwal M., Prakash O., Vivekanand, Pant A.K., Padalia R.C., Mathela S.M. (2012). Essential oil composition and antioxidant activity of leaves and flowers of *Skimmia anquetilia* N.P. Taylor & Airy Shaw. J. Essent. Oil Res..

[18] Kukreti N., Chitme H.R., Varshney V.K. (2023). Antiallergy Activity of *Skimmia anquetilia* on Ovalbumin-induced allergic rhinitis, dermatitis, paw edema and mast cell degranulation. Allergo J. Int..

[19] Annegowda H.V., Bhat R., Min-Tze L., Karim A.A., Mansor S.M. (2012). Influence of sonication treatments and hydro-alcoholic extraction solvents on the phenolics and antioxidants in star fruits. J. Food Sci. Technol..

[20] Elyemni M., Louaste B., Nechad I., Elkamli T., Bouia A., Taleb M., Chaouch M., Eloutassi N. (2019). Hydro-alcoholic extraction of Essential Oils of *Rosmarinus officinalis* L. by Two Different Methods: Hydrodistillation and Microwave Assisted Hydrodistillation. Sci. World J..

[21] Milani G., Curci F., Cavalluzzi M.M., Crupi P., Pisano I., Lentini G., Clodoveo M.L., Franchini C., Corbo F. (2020). Optimization of Microwave-Assisted Hydro-alcoholic extraction of Antioxidants from Bamboo Shoots of *Phyllostachys pubescens*. Molecules.

[22] Sinha S., Raghuwanshi R. (2020). Evaluation of phytochemical, antioxidant and reducing activity in whole plant hydro-alcoholic extract of *Andrographis paniculate* (Burm. f.) Wall. ex Nees. Biosci. Biotechnol. Res. Commun..

[23] Castaldo L., Izzo L., De Pascale S., Narváez A., Rodriguez-Carrasco Y., Ritieni A. (2021). Chemical Composition, In Vitro Bioaccessibility and Antioxidant Activity of Polyphenolic Compounds from Nutraceutical Fennel Waste Hydro-alcoholic extract. Molecules.

[24] Bursal E., Gülçin I. (2011). Polyphenol contents and in vitro antioxidant activities of lyophilised aqueous hydro-alcoholic extract of kiwi fruit (*Actinidia deliciosa*). Food Res. Int..

[25] Al-Rimawi F., Rishmawi S., Ariqat S.H., Khalid M.F., Warad I., Salah Z. (2016). Anticancer Activity, Antioxidant Activity, and Phenolic and Flavonoids Content of Wild *Tragopogon porrifolius* Plant Hydro-alcoholic hydro-alcoholic extracts. Evid.-Based Complement. Altern. Med..

[26] Murugan R., Parimelazhagan T. (2014). Comparative evaluation of different hydro-alcoholic extraction methods for antioxidant and anti-inflammatory properties from *Osbeckia parvifolia* Arn.—An in vito approach. J. King Saud Univ.-Sci..

[27] Kim J., Jang H., Cho W., Yeon S., Lee C. (2018). In vitro antioxidant actions of sulfur-containing amino acids. Arab. J. Chem..

[28] Singhal K.G., Gupta G.D. (2012). Hepatoprotective and antioxidant activity of methanolic hydro-alcoholic extract of flowers of *Nerium oleander* against CCl_4_-induced liver injury in rats. Asian Pac. J. Trop. Med..

[29] Tukappa N.K.A., Londonkar R.L., Nayaka H.B., Kumar C.B.S. (2015). Cytotoxicity and hepatoprotective attributes of methanolic hydro-alcoholic extract of *Rumex vesicarius* L. Biol. Res..

[30] Zhao J., Zhang Z., Dai J., Zhang C., Ye Y., Li L. (2014). Synergistic protective effect of chlorogenic acid, apigenin and caffeic acid against carbon tetrachloride-induced hepatotoxicity in male mice. RSC Adv..

[31] Kanawati G.M., Al-Khateeb I.H., Kandil Y.I. (2021). Arctigenin attenuates CCl_4_-induced hepatotoxicity through suppressing matrix metalloproteinase-2 and oxidative stress. Egypt. Liver J..

[32] Elshopakey G.E., Risha E.F., El-Boshy M.E., Abdalla O.A., Hamed M.F. (2021). Protective effects of thymus vulgaris oil against CCl_4_-mediated hepatotoxicity, oxidative stress and immunosuppression in male albino rats. Adv. Anim. Vet. Sci..

[33] Al-Yahya M., Mothana R., Al-Said M., Al-Dosari M., Al-Musayeib N., Al-Sohaibani M., Parvez M.K., Rafatullah S. (2013). Attenuation of CCl_4_-Induced Oxidative Stress and Hepatonephrotoxicity by Saudi Sidr Honey in Rats. Evid.-Based Complement. Altern. Med..

[34] Domitrović R., Jakovac H., Romić Z., Rahelić D., Tadić Z. (2010). Antifibrotic activity of *Taraxacum officinale* root in carbon tetrachloride-induced liver damage in mice. J. Ethnopharmacol..

[35] Ullah R., Alsaid M., Shahat A., Naser A., Al-Mishari A., Adnan M., Tariq A. (2018). Antioxidant and Hepatoprotective Effects of Methanolic Hydro-alcoholic hydro-alcoholic extracts of *Zilla spinosa* and *Hammada elegans* Against Carbon Tetrachlorideinduced Hepatotoxicity in Rats. Open Chem..

[36] Elgawish R.A.R., Rahman H.G.A., Abdelrazek H.M.A. (2015). Green tea hydro-alcoholic extract attenuates CCl_4_-induced hepatic injury in male hamsters via inhibition of lipid peroxidation and p53-mediated apoptosis. Toxicol. Rep..

[37] Shah M.D., Gnanaraj C., Haque A.T., Iqbal M. (2015). Antioxidative and chemopreventive effects of *Nephrolepis biserrata* against carbon tetrachloride (CCl_4_)-induced oxidative stress and hepatic dysfunction in rats. Pharm. Biol..

[38] Engwa G.A., Ayuk E.L., Igbojekwe B.U., Unaegbu M. (2016). Potential Antioxidant Activity of New Tetracyclic and Pentacyclic Nonlinear Phenothiazine Derivatives. Biochem. Res. Int..

[39] Laouar A., Klibet F., Bourogaa E., Benamara A., Boumendjel A., Chefrour A., Messarah M. (2017). Potential antioxidant properties and hepatoprotective effects of *Juniperus phoenicea* berries against CCl_4_ induced hepatic damage in rats. Asian Pac. J. Trop. Med..

[40] Kherbachı S., Khenıche M., Tacherfıout M. (2022). Antihemolytic activity of hydroalcoholic leaves and bark hydro-alcoholic hydro-alcoholic extracts from Rhamnus alaternus against AAPH induced hemolysis on human erythrocytes. Int. J. Plant Based Pharm..

[41] Rubnawaz S., Okla M.K., Akhtar N., Khan I.U., Bhatti M.Z., Duong H.Q., El-Tayeb M.A., Elbadawi Y.B., Almaary K.S., Moussa I.M. (2021). Antibacterial, Antihemolytic, Cytotoxic, Anticancer, and Antileishmanial Effects of *Ajuga bracteosa* Transgenic Plants. Plants.

[42] Saleh A.A., Elhelbawy N.G., Azmy R.M., Abdelshafy M.S., Donia S.S., Abd El Gayed E.M. (2022). Evaluation of mRNA expression level of the ATP synthase membrane subunit c locus 1 (ATP5G1) gene in patients with schizophrenia. Biochem. Biophys. Rep..

[43] Lopes F.N.C., da Cunha N.V., de Campos B.H., Fattori V., Panis C., Cecchini R., Verri W.A., Pinge-Filho P., Martins-Pinge M.C. (2022). Antioxidant therapy reverses sympathetic dysfunction, oxidative stress, and hypertension in male hyperadipose rats. Life Sci..

[44] Yang C.C., Liao P.H., Cheng Y.H., Chien C.Y., Cheng K.H., Chien C.T. (2022). Diabetes associated with hypertension exacerbated oxidative stress-mediated inflammation, apoptosis and autophagy leading to erectile dysfunction in rats. J. Chin. Med. Assoc. (JCMA).

[45] Ye C., Geng Z., Zhang L.L., Zheng F., Zhou Y.B., Zhu G.Q., Xiong X.Q. (2022). Chronic infusion of ELABELA alleviates vascular remodeling in spontaneously hypertensive rats via anti-inflammatory, anti-oxidative and anti-proliferative effects. Acta Pharmacol. Sin..

[46] Syed A.A., Shafiq M., Reza M.I., Bharati P., Husain A., Singh P., Hanif K., Gayen J.R. (2022). Ethanolic hydro-alcoholic extract of *Cissus quadrangularis* improves vasoreactivity by modulation of eNOS expression and oxidative stress in spontaneously hypertensive rats. Clin. Exp. Hypertens..

[47] Janickova L., Schwaller B. (2020). Parvalbumin-Deficiency Accelerates the Age-Dependent ROS Production in Pvalb Neurons in vivo: Link to Neurodevelopmental Disorders. Front. Cell. Neurosci..

[48] Venkataramaiah C. (2020). Modulations in the ATPases during ketamine-induced schizophrenia and regulatory effect of “3-(3,4-dimethoxy phenyl)-1-(4-methoxyphenyl)prop-2-en-1-one”: An in vivo and in silico studies. J. Recept. Signal Transduct. Res..

[49] Moghaddam A.H., Maboudi K., Bavaghar B., Sangdehi S.R.M., Zare M. (2021). Neuroprotective effects of curcumin-loaded nanophytosome on ketamine-induced schizophrenia-like behaviors and oxidative damage in male mice. Neurosci. Lett..

[50] Wang T., Li C., Han B., Wang Z., Meng X., Zhang L., He J., Fu F. (2020). Neuroprotective effects of Danshensu on rotenone-induced Parkinson’s disease models in vitro and in vivo. BMC Complement. Med. Ther..

[51] Parkhe A., Parekh P., Nalla L.V., Sharma N., Sharma M., Gadepalli A., Kate A., Khairnar A. (2020). Protective effect of alpha mangostin on rotenone induced toxicity in rat model of Parkinson’s disease. Neurosci. Lett..

[52] Bahramikia S., Yazdanparast R. (2010). Antioxidant efficacy of Nasturtium officinale hydro-alcoholic hydro-alcoholic extracts using various in vitro assay systems. J. Acupunct. Meridian Stud..

[53] Wetchakul P., Chonsut P., Punsawad C., Sanpinit S. (2022). LC-QTOF-MS Characterization, Antioxidant Activity, and In Vitro Toxicity of Medicinal Plants from the Tri-Than-Thip Remedy. Evid.-Based Complement. Altern. Med..

[54] Konappa N., Udayashankar A.C., Krishnamurthy S., Pradeep C.K., Chowdappa S., Jogaiah S. (2020). GC-MS analysis of phytoconstituents from Amomum nilgiricum and molecular docking interactions of bioactive serverogenin acetate with target proteins. Sci. Rep..

[55] Süntar I. (2020). Importance of ethnopharmacological studies in drug discovery: Role of medicinal plants. Phytochem. Rev..

[56] Sharma R.K., Sharma N., Kumar U., Samant S.S. (2022). Antioxidant properties, phenolics and flavonoids content of some economically important plants from North-West Indian Himalaya. Nat. Prod. Res..

[57] Giri L., Belwal T., Bahukhandi A., Suyal R., Bhatt I.D., Rawal R.S., Nandi. S.K. (2017). Oxidative DNA damage protective activity and antioxidant potential of *Ashtvarga* species growing in the Indian Himalayan Region. Ind. Crops Prod..

[58] Nabi M., Tabassum N., Ganai B.A. (2022). *Skimmia anquetilia* N.P. Taylor and Airy Shaw (Rutaceae): A Critical Appriasal of its Ethnobotanical and Pharmacological Activities. Front. Plant Sci..

[59] Granato D., Shahidi F., Wrolstad R., Kilmartin P., Melton L.D., Hidalgo F.J., Miyashita K., Camp J.V., Alasalvar C., Ismail A.B. (2018). Antioxidant activity, total phenolics and flavonoids contents: Should we ban in vitro screening methods?. Food Chem..

[60] Mehmood A., Javid S., Khan M.F., Ahmad K.S., Mustafa A. (2022). In vitro total phenolics, total flavonoids, antioxidant and antibacterial activities of selected medicinal plants using different solvent systems. BMC Chem..

[61] Dash P., Ghosh G. (2017). Amino acid composition, antioxidant and functional properties of protein hydrolysates from *Cucurbitaceae* seeds. J. Food Sci. Technol..

[62] Lee Y.S., Cho I.J., Kim J.W., Lee M.K., Ku S.K., Choi J.S., Lee H.J. (2018). Hepatoprotective effects of blue honeysuckle on CCl_4_-induced acute liver damaged mice. Food Sci. Nutr..

[63] Hussain S., Asrar M., Rasul A., Sultana S., Saleem U. (2022). *Chenopodium album* hydro-alcoholic extract ameliorates carbon tetrachloride induced hepatotoxicity in rat model. Saudi J. Biol. Sci..

[64] Shaban N.Z., Awad O.M., Fouad G.M., Hafez A.M., Abdul-Aziz A.A., El-Kot S.M. (2022). Prophylactic and curative effects of Carica papaya Linn. pulp hydro-alcoholic extract against carbon tetrachloride-induced hepatotoxicity in male rats. Environ. Sci. Pollut. Res. Int..

[65] Banwo K., Oduola S., Alao M., Sanni A. (2022). Hepatoprotective potentials of methanolic hydro-alcoholic hydro-alcoholic extracts of Roselle and beetroots against carbon tetrachloride and *Escherichia coli* induced stress in Wistar rats. Egypt. J. Basic Appl. Sci..

[66] Zhao J., Zhang Y., Wan Y., Hu H., Hong Z. (2017). Pien Tze Huang Gan Bao attenuates carbon tetrachloride-induced hepatocyte apoptosis in rats, associated with suppression of p53 activation and oxidative stress. Mol. Med. Rep..

[67] Unsal V., Cicek M., Sabancilar İ. (2020). Toxicity of carbon tetrachloride, free radicals and role of antioxidants. Rev. Environ. Health.

[68] Derouich M., Bouhlali E.D.T., Bammou M., Hmidani A., Sellam K., Alem C. (2020). Bioactive Compounds and Antioxidant, Antiperoxidative, and Antihemolytic Properties Investigation of Three *Apiaceae* Species Grown in the Southeast of Morocco. Scientifica.

[69] Balderrama-Carmona A.P., Silva-Beltrán N.P., Gálvez-Ruiz J.C., Ruíz-Cruz S., Chaidez-Quiroz C., Morán-Palacio E.F. (2020). Antiviral, Antioxidant, and Antihemolytic Effect of *Annona muricata* L. Leaves Hydro-alcoholic hydro-alcoholic extracts. Plants.

