# Supplementary figures and images for: Antioxidant Properties Mediate Nephroprotective and Hepatoprotective Activity of Essential Oil and Hydro-Alcoholic Extract of the High-Altitude Plant Skimmia anquetilia
Source: Antioxidants (Basel). 2023 May 28;12(6):1167. doi: 10.3390/antiox12061167 (PMC10295467; doi:10.3390/antiox12061167)

## Supplementary File

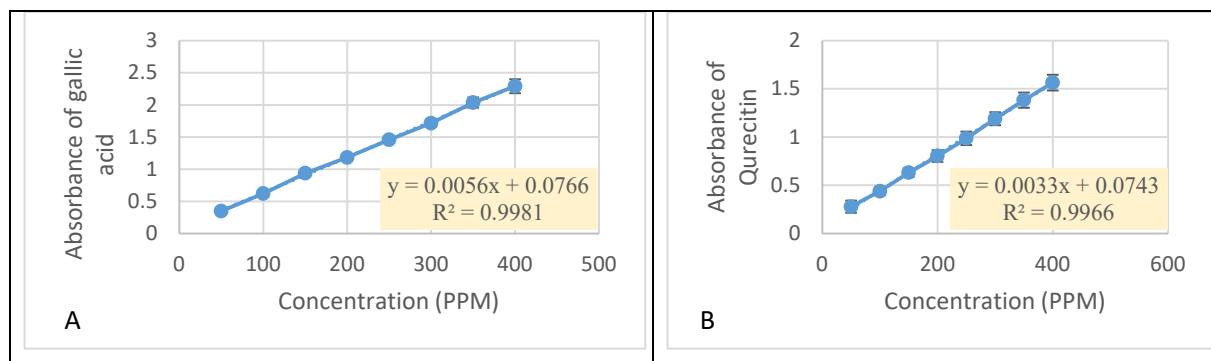

**Figure S1.** A and B showed TPC and TFC of Gallic acid and quercetin. SEM (n=3).

Supplement: Supplementary file 1 [file antioxidants-12-01167-s001.zip › antioxidants-2377981-supplementary.pdf]
